# A Phase III open-label, randomized, active controlled clinical study to assess safety, immunogenicity and lot-to-lot consistency of a bovine-human reassortant pentavalent rotavirus vaccine in Indian infants

**DOI:** 10.1016/j.vaccine.2018.11.006

**Published:** 2018-12-18

**Authors:** Niraj Rathi, Sajjad Desai, Anand Kawade, Padmasani Venkatramanan, Ritabrata Kundu, Sanjay K. Lalwani, A.P. Dubey, J. Venkateswara Rao, D. Narayanappa, Radha Ghildiyal, Nithya Gogtay, P. Venugopal, Sonali Palkar, Renuka Munshi, Gagandeep Kang, Sudhir Babji, Ashish Bavdekar, Sanjay Juvekar, Nupur Ganguly, Prabal Niyogi, Kheya Ghosh Uttam, H.S. Rajani, Alpana Kondekar, Dipti Kumbhar, Smilu Mohanlal, Mukesh C Agarwal, Parvan Shetty, Kalpana Antony, Bhagwat Gunale, Abhijeet Dharmadhikari, Yuxiao Tang, Prasad S. Kulkarni, Jorge Flores

**Affiliations:** a PATH, India; b Serum Institute of India Pvt. Ltd., Pune, India; c Vadu Rural Health Program KEM Hospital Research Centre, Vadu, Pune, India; d Sri Ramachandra Medical Centre, Chennai, India; e Institute of Child Health, Kolkata, India; f Bharati Vidyapeeth Medical College & Hospital, Pune, India; g Maulana Azad Medical College, New Delhi, India; h Gandhi Medical College & Gandhi Hospital, Secunderabad, India; i JSS Medical College & Hospital, Mysore, India; j T.N. Medical College & B.Y.L. Nair Charitable Hospital, Mumbai, India; k Seth GS Medical College & KEM Hospital, Mumbai, India; l Andhra Medical College, Visakhapatnam, India; m The Wellcome Trust Research Laboratory Christian Medical College, Vellore, India; n PATH, USA

**Keywords:** Bovine-Human Reassortant Pentavalent Rotavirus Vaccine (BRV-PV), Lot-to-Lot Consistency, Immunogenicity, Safety

## Abstract

**Background:**

A heat-stable bovine-human rotavirus reassortant pentavalent vaccine (BRV-PV, ROTASIIL®) was developed in India. In this study, the vaccine was tested for safety, immunogenicity and clinical lot-to-lot consistency.

**Methods:**

This was a Phase III, open label, randomized, equivalence design study. The primary objective was to demonstrate lot-to-lot consistency of BRV-PV. Subjects were randomized into four arms, three arms received Lots A, B, and C of BRV-PV and the control arm, received Rotarix®. Three doses of BRV-PV or two doses of Rotarix® and one dose of placebo were given at 6, 10, and 14 weeks of age. Blood samples were collected four weeks after the third dose to assess rotavirus IgA antibody levels. The three lots of BRV-PV were equivalent if the 95% Confidence Intervals (CIs) of the geometric mean concentration (GMC) ratios were between 0.5 and 2. Solicited reactions were collected by using diary cards.

**Results:**

The study was conducted in 1500 randomized infants, of which 1341 infants completed the study. The IgA GMC ratios among the three lots were around 1 (Lot A versus Lot B: 1.07; Lot A versus Lot C: 1.06; and Lot B versus Lot C: 0.99). The 95% CIs for the GMC ratios were between 0.78 and 1.36. The IgA GMCs were: BRV-PV group 19.16 (95% CI 17.37–21.14) and Rotarix® group 10.92 (95% CI 9.36–12.74) (GMC ratio 1.75; 90% CI 1.51–2.04). Seropositivity rates were 46.98% (95% CI 43.86–50.11) and 31.12% (95% CI 26.17–36.41). The incidence of solicited reactions was comparable across the four arms. No serious adverse events were associated with the study vaccines, except two gastroenteritis events in the BRV-PV groups.

**Conclusion:**

Lot-to-lot consistency of BRV-PV was demonstrated in terms of GMC ratios of IgA antibodies. The vaccine safety and immunogenicity profiles were similar to those of Rotarix®.

Clinical Trials.Gov [NCT02584816] and Clinical Trial Registry of India [CTRI/2015/07/006034].

## Highlights

• Pentavalent reassortant rotavirus vaccine (BRV-PV) was tested for lot-to-lot consistency in Indian infants.
• The vaccine demonstrated safety profile similar to licensed Rotarix®.
• Lot-to-lot consistency in the immunogenicity of BRV-PV was demonstrated in terms of GMC ratios of IgA antibodies.
• The immune responses between the pooled BRV-PV arms and Rotarix® were comparable.

## 1 Introduction

Rotavirus gastroenteritis is responsible for significant morbidity and mortality among children under the age of five years, especially in low-resource countries [1]. Over the last decade, rotavirus vaccines have been introduced in many resource-limited countries. Their impact on morbidity and mortality due to rotavirus diseases have been significant [2], [3], [4], [5], [6].

Though two rotavirus vaccines are available internationally (RotaTeq® and Rotarix®), their price remains a constraint, and their availability for world-wide coverage is uncertain. Although the World Health Organization(WHO) recommends universal immunization with rotavirus vaccines [7], only 25% of infants in the world actually receive it [8]. Keeping in mind the global need for affordable vaccines, a new rotavirus vaccine made by bovine-human reassortant technology (BRV-PV) has been developed and tested in India. This heat-stable vaccine was found safe, immunogenic, and efficacious in clinical studies [9], [10], [11], [12] was licensed in 2016 by the Indian Regulatory authorities under the brand name of ROTASIIL® and prequalified by the World Health Organization in September 2018.

As per regulatory requirements the consistency in the production of rotavirus vaccines has to be demonstrated on the basis of titer and physico-chemical characteristics as well as by its immunogenicity in the target population [13]. The present study was undertaken to demonstrate clinical lot-to-lot consistency of BRV-PV in the infants.

## 2 Methods

### 2.1 Ethics

The study was approved by the respective site institutional ethics committees of each of the ten clinical units enrolling the participants, the Western Institutional Review Board (USA), and the Indian regulatory authorities. All subjects were enrolled after their parent(s) gave a written informed consent which was also recorded audio-visually, according to the prevalent Indian regulations. The study population had access to primary health care facilities and referral hospitals.

### 2.2 Study design

This was a Phase III multicentre, open label, randomized, active controlled equivalence study design to test the consistence of the vaccine produced by SIIPL.

Three doses of BRV-PV or two doses of Rotarix^®^ were administered concomitantly with Universal Immunisation Programme (UIP) vaccines (DTwP-HepB-Hib, OPV) four weeks apart. The subjects also received inactivated polio vaccine (IPV) at the age of 14 weeks along with OPV in accordance with the revised UIP. All subjects received trivalent OPV (tOPV) before 25 April 2016 and bivalent OPV (bOPV) following this date. This switch was implemented as per the Global Polio Eradication Initiatives, Polio Eradication Endgame Strategic Plan 2013–2018, approved by the WHO in January 2013 [14].

### 2.3 Selection criteria

The subjects were enrolled from eight urban sites, one semi-urban site, and one rural site. The study population included healthy infants of 6 to 8 weeks of age whose parent(s) provided consent and who had received HepB vaccine and OPV at birth. Presence of diarrhea, vomiting, fever, or any acute disease at the time of enrolment were temporary exclusion criteria. Infants with significant malnutrition or any systemic disorder, congenital abdominal disorders, intussusception, abdominal surgery, impairment of immunological function, persistent diarrhea, or allergy to any components of the study vaccines were excluded from participation in the study.

### 2.4 Investigational products

BRV-PV is a live attenuated, pentavalent human-bovine reassortant rotavirus vaccine (Serum Institute of India Pvt. Ltd., SIIPL). It is available as a lyophilized powder along with 2.5 ml buffered diluent. The powder contains ≥ 10^5.6^ fluorescent focus units (FFU) each of G1, G2, G3, G4, and G9 serotypes. Three batches of BRV-PV (Batch No. 145E40020Z, 145E40030Z, and 145E40040Z, all having an expiration date of December 2017) were used in the study. The buffer diluent contains citrated sodium bicarbonate containing 25.6 gm of sodium bicarbonate and 9.6 gm of citric acid per litre. BRV-PV was administered orally after reconstitution with 2.5 ml of buffer diluent.

Rotarix® (Glaxo SmithKline Biologicals, Belgium) is a live attenuated rotavirus vaccine derived from the human 89–12 strain, which belongs to G1P[8]type. The vaccine used in the study was a lyophilized vaccine to be reconstituted with a liquid diluent in a pre-filled oral applicator. The diluent contains calcium carbonate, xanthan, and sterile water. After reconstitution, each dose of 1 ml contains human rotavirusRIX4414 strain live attenuated not less than 10^6^ CCID_50_. A single batch of Rotarix® was used (Batch No. VAC#XROTA336A1, with an expiration date of January 2018).

Rotarix® was given at 6 and 10 weeks of age, and at 14 weeks, a placebo was administered. The placebo contained lyophilized minimum essential medium (MEM) and excipients to be reconstituted with 2.5 ml of buffer diluent of BRV-PV. A single batch of placebo was used (Batch No. 851E40010Z, with an expiration date of March 2017).

All the subjects received the routine UIP vaccines concomitantly with the study vaccines. They include Pentavac® PFS (DTwP-HepB-Hib vaccine, SIIPL) given by intramuscular injection and BioPolio® (tOPV) (Bharat Biotech International Ltd., India) or BioPolio® (bOPV) (Bharat Biotech International Ltd., India) given orally. In addition, they received one dose of Poliovac PFS® (IPV, SIIPL) given intramuscularly. All the study vaccines except OPV were transported and stored at 2–8 °C, while both bOPV and tOPV were transported and stored at or below −20 °C.

### 2.5 Randomization and blinding

This was an open-label study, however, the laboratory personnel were blinded to the treatment allocation until the analysis was complete. The eligible subjects were randomized to one of the four groups at a ratio of 1:1:1:1 to receive BRV-PV from each of the three lots or Rotarix® according to a computer-generated allocation schedule. The assignments were provided to the sites by a validated interactive web response system (IWRS). A block size of 12 was used to ensure a 1:1:1:1 balance for the study. Different blocks of subject IDs were allocated periodically to each site depending on the enrolment rates. The subject ID allocated to the site was a unique seven-digit alphanumeric number wherein the allocated Randomization ID included A, B, and C, which indicated the three lots of BRV-PV and D indicating Rotarix®/placebo was allotted by the IWRS. There was no restriction on breastfeeding.

## 3 Study outcomes

The study had two primary objectives: to demonstrate manufacturing consistency of BRV-PV by evaluating the immunogenicity of three cGMP lots and to demonstrate the immunological non-inferiority of UIP vaccines when co-administered with the BRV-PV as compared to their co-administration with a licensed rotavirus vaccine. The secondary objectives were: To evaluate the safety of BRV-PV given concomitantly with UIP vaccines and to compare the immunogenicity of BRV-PV vaccine and Rotarix. The lot-to-lot consistency part of the study had an equivalence design while the non-interference with the UIP vaccines was assessed in non-inferiority design [15]. Immune response to Rotarix was also assessed in the study. A total of 1500 subjects were randomized in the study to one of the four groups at a 1:1:1:1 ratio to receive either BRV-PV from one of the three lots (A, B, or C) or Rotarix®.

### 3.1 Immunogenicity assessment

A single blood sample was collected from each child four weeks after the third dose. Clotted blood in the tube was centrifuged to obtain serum. Sera samples were stored at or below −20 °C until the time of analysis. The sera samples were tested for anti-rotavirus immunoglobulin A (IgA) in a validated ELISA conducted at the Wellcome Trust Research Laboratory, Christian Medical College, Vellore, India [16]. Seropositivity was defined as rotavirus specific IgA concentration ≥20 U/ml. In addition, the serum samples were also tested for immune response to each of the EPI vaccines given concomitantly to evaluate any potential interference by BRV-PV. Results from those tests were published separately [15].

### 3.2 Safety assessment

All of the subjects were monitored for 30 min after vaccination for any immediate events. Active surveillance for solicited reactions was maintained for one week after each dose. For that purpose, the parents were given post-immunization diary cards (PIDCs) to record solicited reactions (diarrhea, fever, vomiting, decreased appetite, irritability, and decreased activity level). The study personnel visited each subject’s home after each vaccination to support PIDC recording and to determine the child’s health status.

During the entire period of study, the subjects were monitored for unsolicited adverse events (AEs), intussusception, and serious adverse events (SAEs). Costs of medical management of all AEs were covered by the sponsor regardless of causality or severity of the event.

In addition to the site physicians, a protocol safety review team (PSRT), an intussusception adjudication committee (IAC), and an independent data safety monitoring board (DSMB) provided close monitoring of safety events during the course of the study. While the PSRT comprised of medical officers and statistician from the sponsors and the contract research organization, the DSMB comprised of an independent pediatrician, public health experts, and a biostatistician. The IAC consisted of an independent pediatrician, a pediatric surgeon, and a radiologist to review any potential case of intussusception reported in the study.

### 3.3 Statistical analysis

The full analysis (FA) population included all subjects in the enrolled population who were randomized and received at least one dose of study vaccines and had post-vaccination immunogenicity results. The per protocol (PP) population included all subjects in the FA population who had received all three doses of study vaccines as per the assigned group, within the established vaccination windows and with no major protocol violations. The PP population was the primary analysis population for all immunogenicity objectives, while the FA population results were supportive for immunogenicity objectives. The safety population included all subjects in the enrolled population who received at least one dose of study vaccine and had data for any safety assessment and served as the primary population for safety analysis.

The primary endpoint of the study was geometric mean concentration (GMC) of IgA antibodies after the third dose of BRV-PV. GMCs were compared across Lot A, Lot B, and Lot C groups. Lot consistency was proven if the two sided 95% Confidence Interval (95% CI) for the GMC ratio was within 0.5 and 2.0 for each of the three pair-wise comparisons (A vs. B, A vs. C, and B vs. C). Pair-wise comparisons in terms of proportion of seropositivity were also conducted among the three BRV-PV groups by the Newcombe Hybrid Score method.

Additionally, GMCs and the proportion of seropositivity were also compared between the combined BRV-PV groups and Rotarix® group by the Newcombe Hybrid Score method.

AEs were summarized by number and percentage for each group. All unsolicited AEs and SAEs were coded using MedDRA dictionary version 18.1 and were summarized by System Organ Classification (SOC) and preferred term (PT), severity and relatedness to study vaccines. Medicines received were coded as per the WHO Drug Dictionary and summarized by anatomical therapeutic chemical (ATC) class and WHO drug name (preferred term). For solicited reactions, Fisher’s exact test was used to compare the proportion of subjects among different groups .

The number of evaluable subjects of 300 per lot was necessary to ensure at least 93% overall power to achieve the claim of lot consistency if the true difference among the three lots is up to 1.1 fold and the log_10_ standard deviation is 0.8. Assuming a 20% dropout rate, the required sample size for each lot was 375.

## 4 Results

**Fig. 1:**
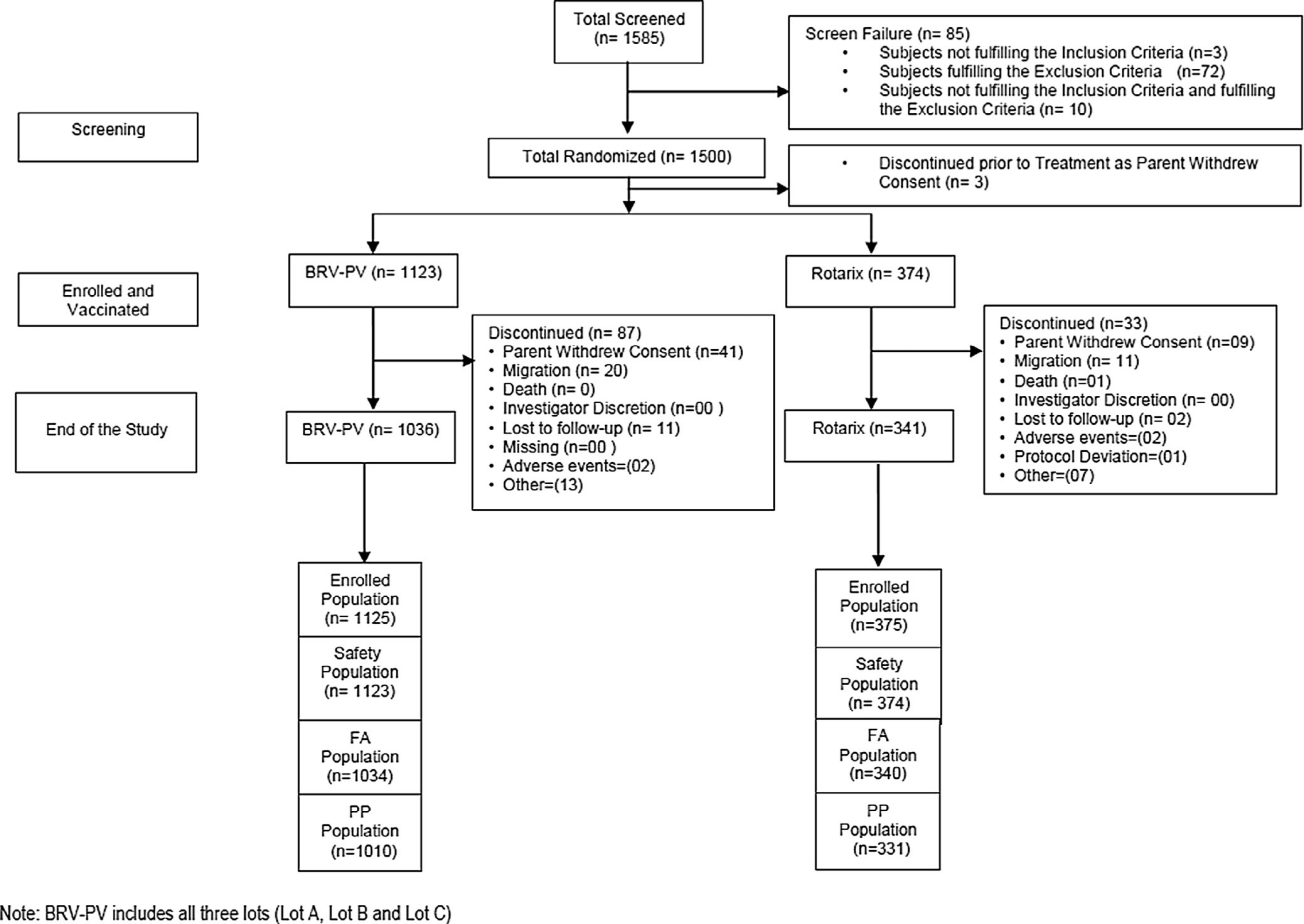
Study flowchart.

A total of 1585 subjects were screened and 1500 eligible subjects were randomized. 1497 subjects received the study vaccine and were part of the safety population. 1374 subjects completed the study and were included in the immunogenicity results, and hence were part of the FA population. Of these, 33 subjects had a major protocol violation and, therefore 1341 subjects were part of the PP population (Fig. 1). The study was conducted across 10 sites in India from December 2015 to November 2016.

The exclusion of 33 subjects from the FA population was because of the following reasons: 20 subjects received vaccines which were not stored appropriately, 10 subjects did not meet eligibility criteria, 2 subjects had received vaccines that were not per the randomization list, and 1 subject received the vaccine and also had blood drawn beyond the allowed window period.

Among the 1500 subjects enrolled, 791 (52.73%) were males. No differences in weight at birth and weight or length at enrolment were observed among the BRV-PV and Rotarix® groups. All subjects had received a birth dose of Hep B and OPV vaccines. The weight at enrolment ranged from 2.5 to 6.6 kg. The length at the time of enrolment ranged from 47 to 63 cm. The age at the time of enrolment ranged from 5.57 to 8.14 weeks (Table 1).

**Table 1:** Demographics and baseline characteristics (Enrolled Population).

Characteristic	BRV-PV Lot A (N = 375)	BRV-PV Lot B (N = 375)	BRV-PV Lot C (N = 375)	BRV-PV Combined (N = 1125)	Rotarix (N = 375)
Male n (%)	202 (53.87)	186 (49.60)	197 (52.53)	585 (52.00)	206 (54.93)
Weight at birth (kg) Mean (SD)	2.82 (0.41)	2.83 (0.43)	2.85 (0.45)	2.83 (0.43)	2.86 (0.42)
Weight at baseline (kg) Mean (SD)	4.27 (0.55)	4.28 (0.59)	4.29 (0.57)	4.28 (0.57)	4.32 (0.55)
Length at baseline (cm) Mean (SD)	54.59 (2.38)	54.57 (2.49)	54.63 (2.49)	54.60 (2.45)	54.83 (2.37)
Age at baseline (weeks) Mean (SD)	6.76 (0.58)	6.72 (0.54)	6.74 (0.55)	6.74 (0.56)	6.78 (0.57)

Of the 1500 subjects randomized, three did not receive any dose because the parents withdrew consent before vaccination. Of the 1497 receiving the first study dose, 84 (5.6%) received only one dose, 19 (1.27%) received only two doses, and 1394 (92.93%) received all three doses. All subjects received DTwP-HepB-Hib and OPV at 6, 10, and 14 weeks concomitantly with BRV-PV or Rotarix®/placebo. All subjects also received IPV at the age of 14 weeks along with OPV as per the current Indian UIP requirements.

24.73% of subjects received tOPV, 42.93% received bOPV, and 32.13% received a combination of tOPV and bOPV concomitantly with BRV-PV and Rotarix® doses. Approximately 72% of the subjects received breastfeeding within 30 min before or after the study vaccination.

### 4.1 Immunogenicity results

For each paired comparison of the BRV-PV vaccine groups, the IgA GMC 95% CI ratios were within the equivalence limits of 0.5 and 2. The lot-to-lot consistency between three consecutive production lots of BRV-PV was thus demonstrated (Table 2). Similar analysis was also conducted for the FA population with the same outcome (not shown).

**Table 2:** GMCs and GMC ratios of Anti-Rotavirus IgA (PP population).

**GMC IgA (U/ml)**			**GMC IgA (U/ml)**			**Ratio of GMCs**	
**Group**	**N**	**Value (95% CI)**	**Group**	**N**	**Value (95% CI)**	**Groups**	**Value (95% CI)**
BRV-PV – Lot A	328	19.98 (16.86–23.67)	BRV-PV – Lot B	337	18.68 (15.73–22.17)	Lot A vs Lot B*	1.07 (0.84–1.36)
BRV-PV – Lot A	328	19.98 (16.86–23.67)	BRV-PV – Lot C	344	18.88 (15.91–22.40)	Lot A vs Lot C*	1.06 (0.83–1.35)
BRV-PV – Lot B	337	18.68 (15.73–22.17)	BRV-PV – Lot C	344	18.88 (15.91–22.40)	Lot B vs Lot C*	0.99 (0.78–1.26)

In terms of IgA seropositivity rates (concentration ≥ 20 u/ml), for each pair of BRV-PV groups, the limits of 95% CI for treatment difference were found within −8.54 and 8.50, thus demonstrating that the three batches were similar by these criteria as well (Table 3). A similar analysis in the FA population supported this finding. The IgA GMCs were 19.16 (95% CI 17.37–21.14) in the combined BRV-PV group and 10.92 (95% CI 9.36–12.74) in the Rotarix® group with a GMC ratio of 1.75 (90% CI 1.51–2.04); this difference was significant (Table 4).

**Table 3:** Anti-Rotavirus IgA seropositivity rates (PP Population).

**Group**	**Seropositive**	**Group**	**Seropositive**	**Difference in seropositivity rates**	
	**n/N, %, 95% CI**		**n/N, %, 95% CI**	**Groups**	**Value (95% CI)**
BRV-PV – Lot A	155/328, 47.26% (41.75–52.82)	BRV-PV – Lot B	156/337, 46.29% (40.87–51.78)	Lot A minus Lot B	0.97 (−6.58 8.50)
BRV-PV – Lot A	155/328, 47.26% (41.75–52.82)	BRV-PV – Lot C	163/344, 47.38% (42.00–52.81)	Lot A minus Lot C	−0.13 (−7.63 7.38)
BRV-PV – Lot B	156/337, 46.29% (40.87–51.78)	BRV-PV – Lot C	163/344, 47.38% (42.00–52.81)	Lot B minus Lot C	−1.09 (−8.54 6.37)

**Table 4:** Comparison of BRV-PV and Rotarix® in terms of IgA seropositivity and GMCs (PP Population).

	BRV-PV combined	Rotarix
Seropositivity n/N, % (95% CI)	474/1009 46.98% (95% CI 43.86–50.11)	103/331 31.12% (95% CI 26.17–36.41)
GMC (IU/ml) (95% CI)	19.16 (95% CI 17.37–21.14)	10.92 (95% CI 9.36–12.74)

### 4.2 Safety results

The safety population comprised of 1497 subjects. There were 11 immediate AEs recorded in the study, all in the BRV-PV group. These consisted of vomiting within five minutes of administration; all were mild and transient. The events were isolated and did not recur in the subsequent doses.

Solicited adverse events (reactogenicity) over the seven days post-vaccination were reported by 940 subjects (83.70%) in the combined BRV-PV groups and 314 subjects (83.96%) in the Rotarix® group. The events were generally mild to moderate in intensity and most resolved within seven days of vaccination. There was no significant difference in reactogenicity rates across the three lots of BRV-PV and between the BRV-PV combined group and Rotarix® (all p > 0.05). All of these subjects had received parallel EPI vaccines, which are known to be reactogenic. Only one subject in the BRV-PV (Lot B) arm was discontinued because of a solicited AE, as he experienced a high fever of 41 °C after the first vaccination (Table 5).

**Table 5:** Incidence of Solicited Adverse Events: after any dose (Safety Population).

Event	BRV-PV Lot A (N = 375) n (%)	BRV-PV Lot B (N = 373) n (%)	BRV-PV Lot C (N = 375) n (%)	Combined BRV-PV (N = 1123) n (%)	Rotarix®^#^ (N = 374) n (%)
Fever	270 (72.00)	280 (75.07)	274 (73.07)	824 (73.37)	273 (72.99)
Irritability	231 (61.60)	227 (60.86)	234 (62.40)	692 (61.62)	222 (59.36)
Decreased appetite	185 (49.33)	171 (45.84)	165 (44.00)	521 (46.39)	157 (41.98)
Decreased activity level	169 (45.07)	162 (43.43)	171 (45.60)	502 (44.70)	164 (43.85)
Vomiting	137 (36.53)	130 (34.85)	131 (34.93)	398 (35.44)	120 (32.09)
Diarrhea	70 (18.67)	54 (14.48)	55 (14.67)	179 (15.94)	77 (20.59)

The distribution of severe solicited AEs was similar in the combined BRV-PV group and the Rotarix® group (16.47% vs. 17.38%; p > 0.05). The incidence of solicited AEs decreased with subsequent doses of BRV-PV and Rotarix® (and EPI vaccines). A total of 1111 subjects (74.21%) after the first dose, 865 subjects (61.22%) after the second dose, and 820 subjects (58.82%) after the third dose had at least one solicited AE. Fever was the most commonly reported solicited event in both the combined BRV-PV (73.37%) and Rotarix® (72.99%) groups. This was followed by irritability, decreased appetite, decreased activity level, vomiting, and diarrhea.

A total of 840 subjects (56.11%) reported 1,924 unsolicited events during the study period. The incidence was similar for both the groups with 628 (55.92%) subjects reporting 1,438 events in the combined BRV-PV group and 212 (56.68%) subjects reporting 486 events in the Rotarix® group (p > 0.05). The most frequently reported event was pain at the DTwP-HepB-Hib injection site: 227 (20.21%) subjects in the combined BRV-PV group and 76 (20.32%) subjects in the Rotarix® group. Other common unsolicited events reported were respiratory tract infection and diarrhea. Out of 1438 events in the BRV-PV group, all except four recovered without any sequelae, two were stabilized, and two were lost to follow-up. Out of the 486 events in the Rotarix® group, all except two were recovered, one was stabilized, and one was fatal.

The majority of these events were mild to moderate. Approximately 4% of the subjects in both groups reported grade 3 events. All events except three in the combined BRV-PV group were unrelated to the study product. Three events of gastroenteritis in the BRV-PV group were considered causally related to the product.

A total of 60 SAEs were reported in 59 subjects in the study. Of these, 40 SAEs were reported by 40 subjects (3.56%) in the combined BRV-PV group and 20 were reported by 19 subjects (5.08%) in the Rotarix® group. There was no significant difference between the groups. Two of the three related unsolicited adverse events led to hospitalization and were also reported as related SAEs. Among the SAEs, there was one death reported in the Rotarix® group. This was caused by sepsis and was causally unrelated to the study vaccine. No case of intussusception was reported in the study.

## 5 Discussion

This Phase III, multicenter, open-label, randomized study evaluated lot-to-lot consistency of BRV-PV in 6–8-week-old infants at 10 sites in India. Three doses of BRV-PV or two doses of Rotarix® were administered concomitantly with UIP vaccines, four weeks apart. Clinical lot-to-lot consistency was clearly established by meeting the typical pre-specified statistical criteria set in advance.

The IgA seropositivity rates with BRV-PV in this study were around 47%. In the previous Phase II study, this rate was 56.67% among children receiving vaccine and 11.54% among placebo recipients [9]. These results are similar to those reported for Rotarix® in an Indian study where the seroconversion rates were 58.3% [95% CI: 48.7; 67.4] in the Rotarix® group and 6.3% [95% CI: 2.5; 12.5] in the placebo group [17]. An Indian study on the 116E rotavirus vaccine showed 89.7% seroconversion in the vaccine arm and 28.1% in the placebo arm [18]. The Indian study of RotaTeq® showed 83% three-fold rise (seroconversion) in serum IgA antibodies, however the study did not include a placebo arm [19]. A recent study in India also showed seropositivity rates ranging from 38.3% (95%CI: 32.8, 43.9) to 42.1% (95%CI: 36.6, 47.9), with three different formulations of Rotavac [20].

BRV-PV was well tolerated with very few incidents of vomiting as immediate AEs. The combined BRV-PV groups were similar to Rotarix® in terms of solicited symptoms reported during the 7-day solicited period and unsolicited symptoms reported during the 28-day follow-up period. The study was not powered for safety comparisons between the two vaccines. A full safety evaluation of the vaccine was conducted in previous studies in India and Niger [11], [12].

The majority of unsolicited events were mild to moderate, and all the events except for three gastroenteritis cases in the combined BRV-PV group were considered to be unrelated to the study product. No confirmation of their association with the vaccine could be established, since stool specimens were not available for testing. No intussusception cases were reported in this study.

The seropositivity rate as determined 4 weeks after the last vaccination was higher for BRV-PV than that of Rotarix® (46.98% vs. 31.12%), and the GMC for the combined BRV-PV groups was also higher as compared to Rotarix® (19.16 vs. 10.92 U/ml). Although BRV-PV appeared to be more immunogenic than Rotarix®, the differences observed could be due to other factors such as antigen used in the IgA (the antigen in the ELISA test was from a bovine rotavirus strain), and timing of sampling. Thus, the control group received two doses of Rotarix® at 6 and 10 weeks of age with placebo administration at 14 weeks or to blood sampling window after last dose, i.e., the blood sample for immunogenicity was withdrawn four weeks after last vaccination with BRV-PV whereas it was withdrawn eight weeks after last vaccination with Rotarix®. Baseline antibody levels were not measured, however, the fact that the study was randomized ensures that prior exposure to rotavirus must have been similar among the four study groups.

To conclude, lot-to-lot consistency of three lots of BRV-PV has been demonstrated. The vaccine was also found to have a similar safety profile as Rotarix®.

## Conflict of interest

Dr. Prasad S. Kulkarni, Dr. Sajjad Desai, Dr. Bhagwat Gunale, and Mr. Abhijeet Dharmadhikari are employed by SIIPL, which manufactures the BRV-PV.
